# Mucosa-Associated Lymphoid Tissue Lymphoma Translocation Protein 1 Positively Modulates Matrix Metalloproteinase-9 Production in Alveolar Macrophages upon Toll-Like Receptor 7 Signaling and Influenza Virus Infection

**DOI:** 10.3389/fimmu.2017.01177

**Published:** 2017-09-22

**Authors:** Yu-Hsiang Lee, Juin-Hua Huang, Tzu-Hsuan Chang, Hung-Chih Yang, Betty A. Wu-Hsieh

**Affiliations:** ^1^Graduate Institute of Immunology, College of Medicine, National Taiwan University, Taipei, Taiwan; ^2^Graduate Institute of Microbiology, College of Medicine, National Taiwan University, Taipei, Taiwan

**Keywords:** alveolar macrophages, matrix metalloproteinase-9, toll-like receptor 7, mucosa-associated lymphoid tissue lymphoma translocation protein 1, influenza A virus, pulmonary inflammation

## Abstract

Influenza A virus (IAV) infection causes significant morbidity and mortality worldwide. Matrix metalloproteinase-9 (MMP-9) degrades extracellular matrix and is involved in the pathology of influenza. It has been reported that MMP-9 mediates neutrophil migration in IAV infection. Whether alveolar macrophages, the first immune cells that encounter IAV, produce MMP-9, and the mechanism of its regulation have never been investigated. As Toll-like receptor 7 (TLR7) is one of the receptors in innate immune cells that recognize IAV, we used TLR7 agonists and IAV to stimulate alveolar macrophage MH-S cells, primary macrophages, and bone marrow neutrophils. Results showed that MMP-9 expression in macrophages is inducible by TLR7 agonists and IAV, yet, MMP-9 production by neutrophils is not inducible by either one of them. We hypothesized that MMP-9 production in macrophages is mediated through TLR7-NF-κB pathway and used microarray to analyze TLR7 agonist-induced NF-κB-related genes. Mucosa-associated lymphoid tissue lymphoma translocation protein 1 (MALT1), a positive regulator of NF-κB, is amongst the top highly induced genes. By use of MALT1 inhibitor (z-VRPR-fmk) and alveolar macrophages from MALT1-deficient mice, we found that MMP-9 production is MALT1-dependent. While MALT1 can act as a paracaspase in lymphocytes through degrading various signaling proteins, we discovered that MALT1 functions to reduce a negative regulator of NF-κB, cylindromatosis (CYLD), in alveolar macrophages. IAV-induced MMP-9, TNF, and IL-6 in lungs of MALT1-deficient mice are significantly lower than in wild-type mice after intratracheal infection. MALT1-deficient mice also have less body weight loss and longer survival after infection. Taken together, we demonstrated a novel role of MALT1 in regulating alveolar macrophage MMP-9 production whose presence exacerbates the severity of influenza.

## Introduction

Influenza virus causes 1 billion infections and 300,000–500,000 deaths worldwide each year (1). Although anti-influenza drugs have been approved to use and are effective, some influenza A virus (IAV) isolates are demonstrated to be drug resistant (1). Understanding host response to influenza may be an alternative strategy that circumvents drug resistance to treat influenza disease.

Matrix metalloproteinases (MMPs) are endopeptidases that regulate tissue remodeling by degrading extracellular matrix (2). Matrix metalloproteinase-9 (MMP-9) (also known as gelatinase-B) is known to exacerbate acute lung injury and chronic pulmonary disorder (3, 4). Importantly, MMP-9 upregulation is associated with viral infection-induced tissue injury, including IAV infection (5–7). In a mouse model of IAV infection, MMP-9 is upregulated in the lungs (8). By comparing infection by highly lethal strain of IAV to mild strain in the mouse, we observed that MMP-9 upregulation correlates with severe pathology in infection by highly lethal IAV and that inhibiting MMP-9 partially rescues IAV-induced lung pathology (9). Thus, MMP-9 is important to the pathogenesis of influenza. Bradley et al. reported that IAV infection induces neutrophil infiltration to the lungs and neutrophils produce MMP-9 (10). However, MMP-9 is not upregulated in human neutrophils stimulated by IAV (11). These results suggest that neutrophil MMP-9 production is not regulated by virus *per se*. Whether MMP-9 production by other innate immune cells, i.e., macrophages, is regulated still remains a question to be addressed.

A number of studies showed that alveolar macrophages play a protective role in IAV infection (12–14). In granulocyte-macrophage colony-stimulating factor-deficient mice, which lack alveolar macrophages and in WT mice selectively depleted of alveolar macrophage, there is impaired gas exchange, more body-weight loss, and higher mortality after IAV infection (12). It is recently shown that conditional depletion of alveolar macrophages increases infection of type I alveolar epithelial cells by IAV, but it does not affect IAV clearance nor the establishment of anti-IAV adaptive immune responses (14). Alveolar macrophages are known to contribute to the pathogenesis in respiratory infection by human metapneumovirus while they are being protective in respiratory syncytial virus infection (15). It remains to be clarified whether alveolar macrophages in IAV infection also play a pathogenic role.

Toll-like receptor 3 (TLR3) is known to recognize IAV in human respiratory epithelial cells whereas TLR7 detects IAV RNA in the endosomes of mouse macrophages (16, 17). By use of knockout mice, it is shown that IAV-induced MMP-9 response in bronchoalveolar lavage (BAL) cells is MyD88- and TLR3-dependent (10). MyD88- and TLR3-deficiency reduces MMP-9-producing cell number by 50 and 20%, respectively (10). These data indicate that in addition to TLR3, there is another pathway(s) that may be involved in MMP-9 production in IAV infection. We reasoned that as alveolar macrophages are sentinel cells in the lungs, they are likely the first immune cells to encounter IAV. TLR7 signaling pathway in alveolar macrophages may play an important role in their response to the virus.

It is reported that MMP-9 production is mainly regulated by NF-κB signaling pathway (18, 19). Caspase activation and recruitment domain (CARD)/B-cell lymphoma 10 (BCL10)/mucosa-associated lymphoid tissue lymphoma translocation protein 1 (MALT1) complex (CBM) complex, which acts upstream to promote NF-κB activation, is composed of MALT1, BCL10, and (CARD)-containing protein. While CBM complex in lymphoid cells is composed of CARD11 (also known as CARMA1), myeloid-type CBM complex is of CARD9 (20, 21). In lymphocytes, MALT1 regulates NF-κB signaling through a scaffold function that transduces signals from CBM complex to TRAF6, which subsequently activates downstream signaling (20). As a paracaspase, MALT1 also functions to cleave substrate proteins in activated lymphocytes (22). However, the mechanism of how MALT1 regulates NF-κB signaling in myeloid cells is still poorly understood.

We aimed to study the regulation of MMP-9 production in alveolar macrophages. Since TLR7 is one of the major receptors that recognize IAV in macrophages (17), we used TLR7 agonists and IAV to stimulate alveolar macrophage to induce MMP-9 production. Employing knockout mice and inhibitors, we tested the hypothesis that MALT1 modulates TLR7-NF-κB signaling in alveolar macrophages, thereby regulates the subsequent cellular responses including that of MMP-9 production to affect the severity of IAV infection. Our study revealed a novel role of MALT1 in regulating alveolar macrophage MMP-9 production and showed that MALT1 deficiency alleviates the severity of influenza.

## Materials and Methods

### Mice

Mucosa-associated lymphoid tissue lymphoma translocation protein 1-deficient mice were originally obtained from Dr. Tak W. Mak (Campbell Family Institute for Breast Cancer Research, University Health Network, Canada) and bred in National Laboratory Animal Center (NLAC, Taiwan). *Malt1*-deficient clone was isolated from a 129/J library (23) and injected into embryonic stem cells from C57BL/6 mice. The progeny were crossed back to C57BL/6 to generate homozygous *Malt1^−/−^* mice in C57BL/6 background (24). C57BL/6 mice were purchased from NLAC (originally from the Jackson Laboratory) and maintained in National Taiwan University College of Medicine Laboratory Animal Center (NTU CMLAC). All mice were housed in filter-top cages under specific pathogen-free conditions. This study was approved by the Institutional Animal Care and Use Committee of the National Taiwan University College of Medicine and College of Public Health (IACUC No. 20140314). All experiments were carried out in strict accordance to the Guidebook for the Care and Use of Laboratory Animals, The Third Edition, 2007, published by The Chinese-Taipei Society of Laboratory Animal Sciences (Taipei, Taiwan). All infection experiments in mice were performed following the guidelines of biosafety level 2.

### Cells

MH-S cells, a murine alveolar macrophage cell line, were purchased from Bioresource Collection and Research Center (BCRC, Taiwan) and maintained in complete RPMI 1640 medium (ThermoFisher, MA, USA) containing 10% FBS (Biological Industries, CT, USA). Primary alveolar macrophages were isolated from BAL. Briefly, 1 ml of ice-cold sterile saline solution containing 0.6 mM of EDTA was injected into mouse lungs and fluid was aspirated. The procedure was repeated five times. Alveolar macrophages (CD11c^+^Siglec-F^+^) constituted 97 ± 0.3% of the total lavage cells. To obtain neutrophils, bone marrow cells were harvested from the femurs of mice and suspended in DPBS before overlaid on discontinuous percoll gradients (55, 62, and 81%) (GE healthcare, PA, USA). Cells were centrifuged at 1,400 × *g* for 30 min. Cells at the interface between 62 and 81% fractions were harvested. Neutrophils (CD11b^+^Ly6G^+^) constituted 92% of the total cells harvested. Thioglycollate-elicited peritoneal macrophages were harvested from wild-type mice. Mice were injected with 1 ml of 3% thioglycollate (Sigma) intraperitoneally, and peritoneal cells were collected on day 4 after injection. Cells were cultured overnight, and the monolayers were washed three times with Hank’s Balanced Salt Solution (Biological Industries) to remove non-adherent cells. Adherent cells were used in experiments.

### Viral Propagation, Infection, and Inactivation

Influenza A/WSN/33 virus (H1N1) and A/HKx31 (H3N2) were propagated in Madin-Darby canine kidney (MDCK) cells in serum-free Dulbecco’s Modified Eagle Medium supplemented with TPCK-trypsin (2 µg/ml), which cleaves hemagglutinin of IAV and HEPES (10 mM) (infectious medium). The viral titer was determined by plaque assay in MDCK cells. For intratracheal inoculation, mice were anesthetized by intraperitoneal injection of Tribromoethanol (250 mg/kg, Avertin). To ensure accurate delivery, IAV at indicated titer in infectious medium was inoculated into the trachea through a small incision, and the cut was sutured after inoculation. Sham control mice were inoculated through the same route with the same volume of infectious media. To inactivate virus, virus containing supernatant was placed in 60 mm Petri dish with fluid depth of 10 mm. Dish placed on ice was exposed to 4,000 ergs cm^−2^ from a UV source for 45 min.

### TLR Agonists and Inhibitors

Lipopolysaccharides (LPS, TLR4 agonist) was purchased from Sigma-Aldrich. Both poly I:C (TLR3 agonist) and imiquimod (R837, TLR7 agonist) were from InvivoGen; R848 (TLR7/8 agonist) and z-VRPR-fmk (MALT1 inhibitor) were from Enzo Life Science (NY, USA). JSH-23 (NF-κB activation inhibitor II) was obtained from Merck Millipore and SR11302 [activator protein 1 (AP-1) inhibitor] was from TOCRIS (Bristol, UK).

### ELISA

ELISA kits to determine the concentration of surfactant protein-D (SP-D) in BAL and culture supernatants were from Sino Biological (Beijing, China). ELISA kit to quantify MMP-9 was from R&D Systems (MN, USA), that for interleukin-6 (IL-6) and tumor necrosis factor (TNF) were rom eBioscience (MA, USA). All experimental procedures followed the manufacturer’s instructions.

### Microarray

Total RNA was extracted from MH-S cells stimulated with or without R848 (10 µM) using RNeasy kit (QIAGEN, CA, USA). Extracted RNA was applied to Mouse OneArray chips (Phalanx, Taiwan). Fold-change was calculated by Rosetta Resolver 7.2 with error model adjusted followed by Amersham Pairwise Ration Builder for signal comparison. The experiment was performed with two technical repeats for one biological sample. The following are accession numbers of genes: Malt1: NM_172833.2, TNFAIP3: NM_009397.3, IKBKE: NM_019777.3, Card9: NM_001037747.1, Atf1: NM_007497.3, JUN: NM_010591.2, MAP3K1: NM_011945.2, BCL2L1: NM_009743.4, TRAF6: NM_009424.2, IRAK1: NM_001177973.1, PSIP1: NM_133948.4.

### Quantitative Real-time PCR (qPCR)

Total mRNA was extracted from MH-S cells with or without stimulation as well as from sorted or total BAL cells from mice with or without IAV infection using Quick-RNA MiniPrep kit (Zymo Research, CA, USA). Extracted RNA was reversely transcribed to cDNA by using Superscript III Reverse Transcriptase (Invitrogen, MA, USA). cDNA was amplified in a 10 µl reaction mixture containing primers and Fast SYBR Green Master Mix (Applied Biosystems, CA, USA) in PikoReal96 Real-time PCR detection system (ThermoFisher, MA, USA). The condition for amplification was denaturation at 95°C for 3 min, followed by 40 cycles of 95°C for 5 s, 60°C for 20 s, and 1 cycle of 60°C for 30 s. The expression of *Malt1, Mmp9*, and viral nucleoprotein (NP) transcripts were normalized against *Actb*. The sequences of primers for *Malt1* gene were 5′-CGC AGA GTT CTC CAA TGT CA-3′ and 5′-GAG TCC CCT TGT TTG CAT GT-3′; for *Mmp9* gene, 5′-CTG GAC AGC CAG ACA CTA AAG-3′ and 5′-CTC GCG GCA AGT CTT CAG AG-3′ and for *Actb* gene, 5′-TGT ATG AAG GCT TTG GTC TCC CT-3′and 5′-AGG TGT GCA CTT TTA TTG GTC TCA A-3′; for NP gene, 5′-GAT TGG TGG AAT TGG ACG AT-3′ and 5′-AGA GCA CCA TTC TCT CTA TT-3′.

### Western Blotting

Cells were lysed in PhosphoSafe™ Extraction Reagent (Merck Millipore, MA, USA) and boiled in TOOLS SDS-PAGE loading buffer (BIOTOOLS, Taipei, Taiwan). Cell lysates were analyzed on 10% sodium dodecyl sulfate polyacrylamide gel. Proteins were detected by immunoblotting using primary antibodies against MALT1 (Santa Cruz, TX, USA), Cylindromatosis 1 (CYLD) (Santa Cruz), regnase-1 (gift of Dr. Shizuo Akira) (25), RelB (Cell signaling, MA, USA), p50 (Santa Cruz), p65 (Santa Cruz), phospho-c-Jun (Abcam), phospho-c-Fos (Cell signaling), lamin A+C (Abcam, MA, USA), and beta-actin (GeneTex, Hsinchu, Taiwan). Horseradish peroxidase-conjugated goat anti-rabbit (GeneTex) and rabbit anti-mouse (GeneTex) antibodies were used as secondary antibodies. The intensity of the blots was quantified by ImageJ™ software (NIH, USA).

### Nucleus/Cytosol Fractionation

MH-S cells (1.5 × 10^6^) were stimulated with or without imiquimod (1.0 µg/ml) and harvested by scraper. Cells were suspended in 40 µl of hypotonic buffer (10 mM Tris–HCl, 10 mM NaCl, 3 mM MgCl_2_, 0.5% NP-40, pH 7.4) with repeated pipetting. After being left on ice for 3 min, cells were centrifuged at 6,000 × *g* at 4°C for 5 min. The supernatant was saved as cytosol fraction. The pellet was washed with 200 µl of hypotonic buffer and resuspended in 40 µl of PhosphoSafe™ Extraction Reagent (Merck Millipore). After centrifugation at 13,000 × *g* at 4°C for 5 min, the supernatant was collected as the nuclear fraction and stored at −80°C until analysis.

### BAL Cell Phenotyping and Sorting

Cells in BAL were stained with FITC-conjugated rat anti-mouse F4/80 (eBioscience), PE-conjugated rat anti-mouse Siglec-F (BD Biosciences, CA, USA), APC-conjugated hamster anti-mouse CD11c (eBioscience) and APC-conjugated rat anti-mouse Ly6G (BioLegend, CA, USA) antibodies. For phenotyping, stained cells were analyzed by FACSCanto II (BD Biosciences). Stained cells were sorted by FACSAria (BD Biosciences) through the service provided by the Flow Cytometric Analyzing and Sorting Core (the First Core Laboratory, National Taiwan University College of Medicine).

### Total Protein Assay

Total protein in cell-free BALs was quantified by Bradford assay (Bio-Rad, CA, USA). Absorbance was read at 595 nm and compared to bovine serum albumin standards.

### Hematoxylin and Eosin Stain

Killed mice were intratracheally inoculated with 0.7 ml of 10% formalin solution for tissue fixation. Lung tissues were embedded in paraffin wax and the sections were stained with hematoxylin and eosin stain.

### LDH Cytotoxicity Assay

Percent cell death was quantified by CytoTox 96^®^ Non-Radioactive Cytotoxicity Assay (Promega, WI, USA). Briefly, CytoTox 96^®^ Reagent was added to culture supernatants and to total cell lysates. The mixture was incubated at room temperature for 30 min. The absorbance signal was measured at 490 nm in a plate reader after stop solution was added.

### Statistical Analysis

The comparisons between two groups were analyzed by non-parametric Mann–Whitney *U*-test. The comparisons among multiple groups were analyzed by ANOVA followed by Sidak’s, Tukey’s, Dunnett’s, or non-parametric Kruskal–Wallis multiple comparisons test. The tests used for statistical analysis were specified in figure legends. Survival was analyzed by Log-rank test. All statistical tests were performed by GraphPad Prism 6.01 (GraphPad Software, CA, USA).

## Results

### MALT1 Positively Regulates IAV- and TLR7 Agonist-Induced MMP-9 Production in Alveolar Macrophages

**Figure 1:**
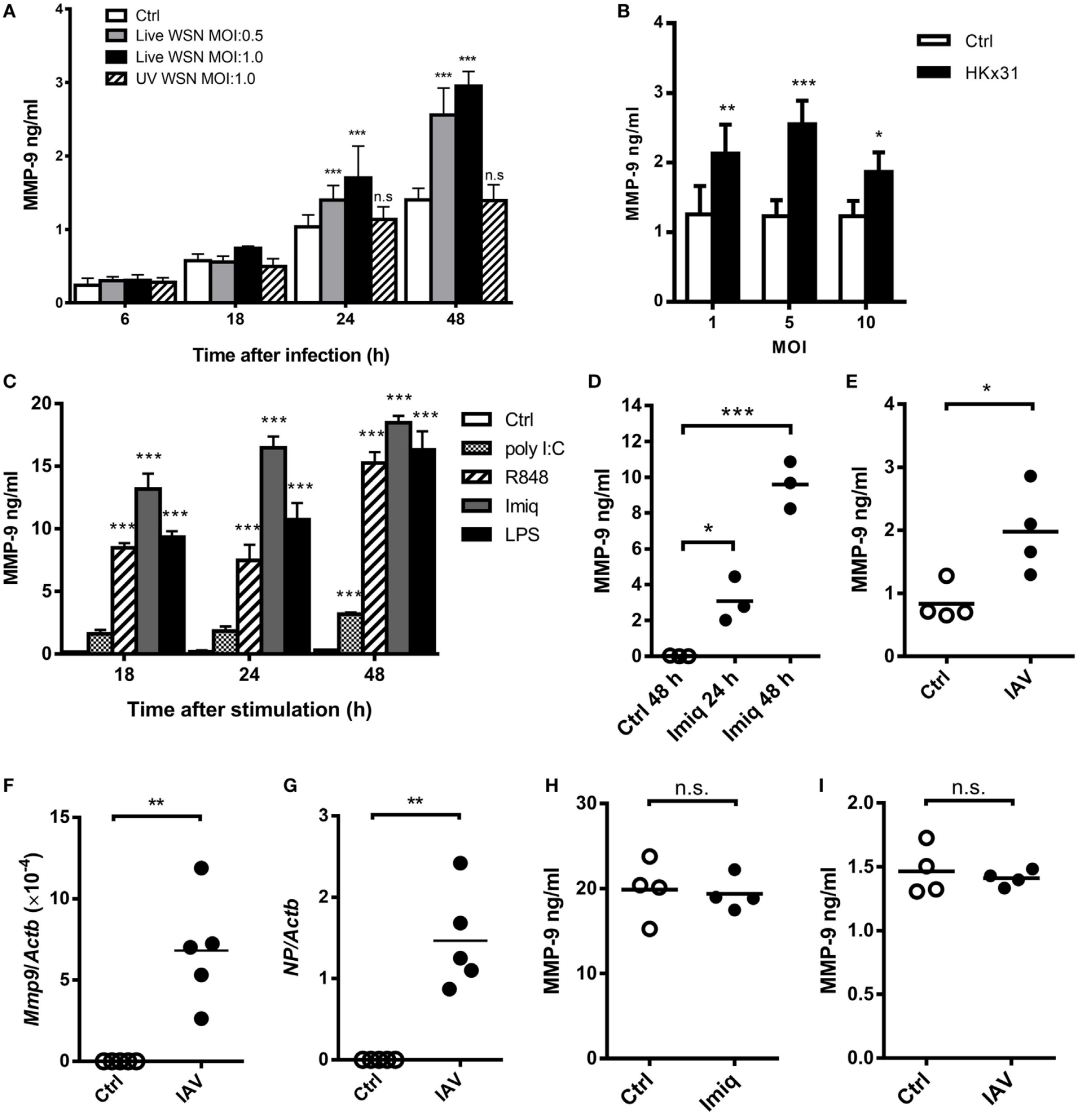
Influenza A virus (IAV) and toll-like receptor 7 agonists induce alveolar macrophage matrix metalloproteinase-9 (MMP-9) production. **(A)** MH-S cells were stimulated with or without live or UV-inactivated WSN strain of IAV at different MOIs for indicated periods of time (*n* = 4, four independent experiments). **(B)** MH-S cells were stimulated with or without HKx31 virus at different MOIs for 48 h before harvest (*n* = 4, four independent experiments). **(C)** MH-S cells were stimulated with or without poly I:C (50 µg/ml), R848 (10 µM), imiquimod (Imiq, 1 µg/ml), or LPS (1 µg/ml) for indicated periods of time (*n* = 3, three independent experiments). **(D)** Primary alveolar macrophages of wild-type mice stimulated with or without Imiq (1 µg/ml) for indicated periods of time. **(E)** Thioglycollate-elicited peritoneal macrophages stimulated with or without HKx31 virus (MOI = 10) for 48 h. **(F,G)** Wild-type mice were infected with 5 × 10^3^ PFU of WSN virus intratracheally for 2 days. Alveolar macrophages (Siglec-F^+^F4/80^+^) in bronchoalveolar lavage were sorted and *Mmp9* and viral nucleoprotein mRNA were quantified by qPCR and normalized against *Actb*. Primary neutrophils from bone marrow were stimulated with or without **(H)** Imiq (1 µg/ml) and **(I)** WSN virus (MOI = 1) for 24 h. **(D–I)** Each dot represents cells from one mouse. **(A–E,H,I)** MMP-9 in culture supernatants was quantified by ELISA. Data presented are the mean + SD. *p < 0.05, **p < 0.01, and ***p < 0.001 when compared to unstimulated cells. **(A–D)** was analyzed by one-way ANOVA, followed by **(A)** Turkey’s, **(B)** Sidak’s, **(C,D)** Dunnett’s *post hoc* tests, and **(E–I)** by Mann–Whitney *U*-test.

It has been demonstrated that acute lung injury induced by IAV infection, LPS, and phorbol myristate acetate stimulation is positively associated with MMP-9 production (8, 9, 26). TLR7 is an important receptor to recognize IAV in innate cells (27). To investigate the regulation of IAV-induced MMP-9 production, we first determined whether stimulation by different strains of IAV and TLR agonists induces MMP-9 production in macrophages. Murine alveolar macrophage MH-S cells were stimulated with WSN and HKx31 virus as well as poly I:C, imiquimod, R848, and LPS. Results showed that viable WSN and HKx31 virus but not UV-inactivated IAV induced MMP-9 production (Figures 1A,B). MMP-9 production was low at MOI of 10 when about 65% of cells were dead (Figure 1B; Figure S1A in Supplementary Material). TLR7 agonists imiquimod, R848, TLR3 agonist poly I:C, and TLR4 agonist LPS induced MMP-9 production across all time points studied (Figure 1C). Primary alveolar macrophages stimulated by imiquimod and peritoneal macrophages infected by IAV (HKx31) also produced MMP-9 (Figures 1D,E). Additionally, alveolar macrophages sorted from IAV (WSN)-infected mice expressed both viral *NP* and *Mmp-9* transcripts (Figures 1F,G). Interestingly, primary neutrophils produced MMP-9 without stimulation. Stimulation by either imiquimod (Figure 1H) or WSN virus (Figure 1I) did not enhance MMP-9 production. These results together showed that different IAVs and TLR agonists induce the production of MMP-9 by macrophages.

**Figure 2:**
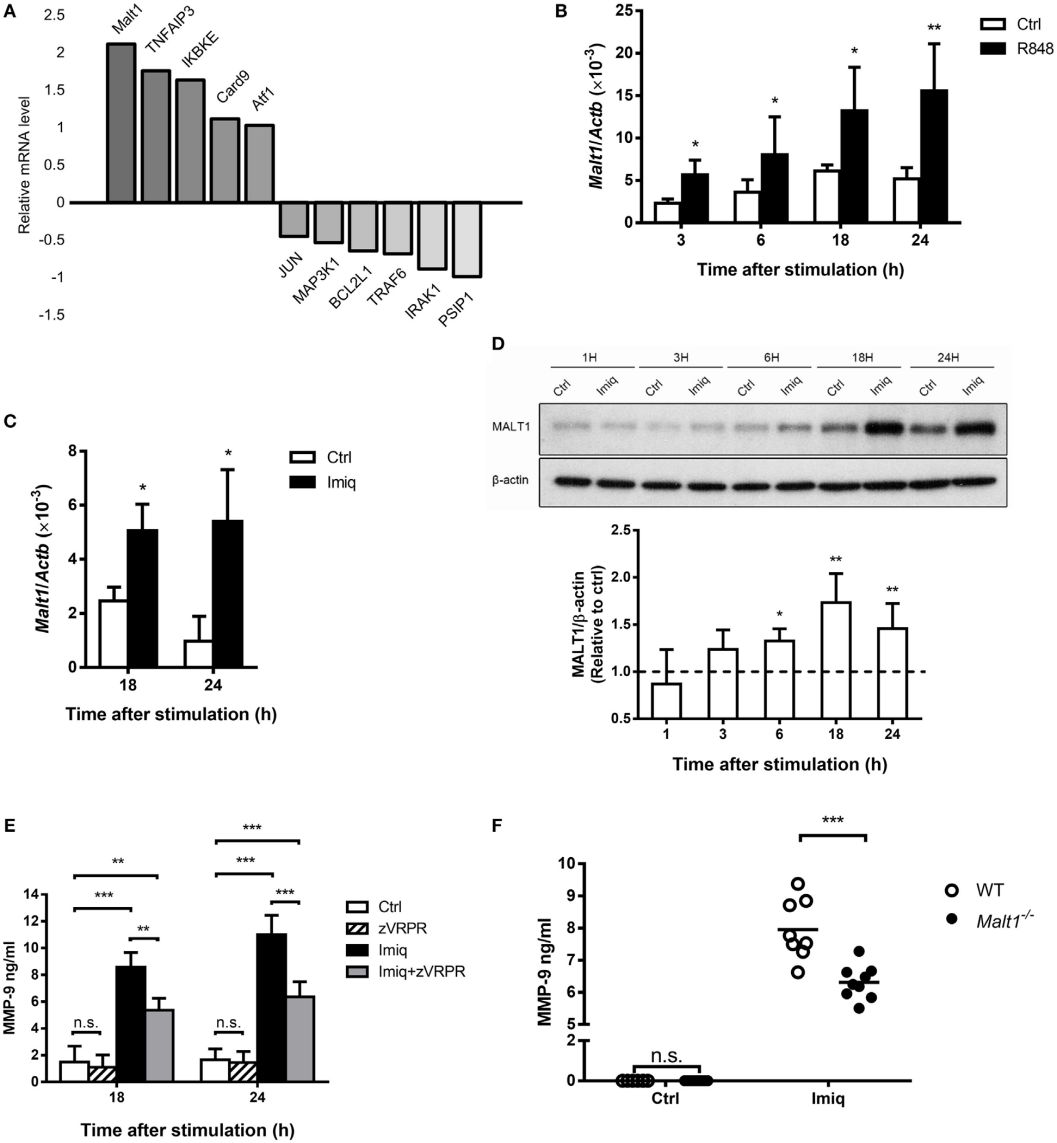
Toll-like receptor 7-mediated mucosa-associated lymphoid tissue lymphoma translocation protein 1 (MALT1) upregulation is required for matrix metalloproteinase-9 (MMP-9) production in alveolar macrophages. **(A)** MH-S cells were stimulated with or without R848 (10 µM). RNA was extracted at 24 h after stimulation and applied to microarray chip. Relative levels of mRNA expression were compared to unstimulated cells. Data were compiled from one experiment. **(B,C)** MH-S cells were stimulated with or without **(B)** R848 (10 µM) and **(C)** imiquimod (Imiq, 1 µg/ml). The level of *Malt1* mRNA was quantified by qPCR and normalized against *Actb* at indicated time points (*n* = 4, four independent experiments). **(D)** MH-S cells were stimulated with or without Imiq (1 µg/ml) for indicated periods of time. Cell lysates were analyzed for MALT1 and β-actin protein by Western blotting. Bar graphs show the levels of MALT1 protein normalized against β-actin (*n* = 4, four independent experiments. One representative experiment is shown). The MALT1-to-β-actin ratio at each time point without stimulation (Ctrl) was taken as 1.0. **(E)** MH-S cells were pretreated with z-VRPR-fmk (zVRPR, MALT1 inhibitor, 100 µM) for 6 h before stimulation with or without Imiq (1 µg/ml) for another 18 and 24 h. MMP-9 concentration was quantified by ELISA (*n* = 3, three independent experiments). **(F)** Primary alveolar macrophages from wild-type and MALT1-deficient mice were stimulated with or without Imiq (1 µg/ml) for 48 h. The concentration of MMP-9 in culture supernatants was quantified by ELISA. Each dot represents cells from one mouse and data are a compilation of three independent experiments. n.s., not significant, *p < 0.05, **p < 0.01, and ***p < 0.001 compared to control. **(B–D)** were analyzed by Mann–Whitney *U*-test, and **(E,F)** were analyzed by one-way ANOVA followed by **(E)** Tukey’s and **(F)** Sidak’s *post hoc* tests.

To investigate the regulation of MMP-9 by TLR7 signaling in macrophages, we stimulated MH-S cells with TLR7/8 agonist R848 (28) and compared their gene expression profile to unstimulated cells by microarray analysis. A number of NF-κB-related genes were up- and downregulated after stimulation (Figure 2A). *Malt1* mRNA induction was the highest among all the upregulated genes (Figure 2A). Quantitative PCR results confirmed that stimulation by TLR7/8 agonists R848 (Figure 2B) and TLR7 agonist imiquimod (Figure 2C) upregulated *Malt1* mRNA and that MALT1 protein expression was significantly upregulated at 18 and 24 h after stimulation (Figure 2D). Inhibition of MALT1 activity by z-VRPR-fmk significantly reduced MMP-9 production after imiquimod stimulation (Figure 2E). Moreover, alveolar macrophages deficient in MALT1 also produced significantly less MMP-9 upon stimulation by imiquimod (Figure 2F). Taken together, we demonstrated that MALT1 positively regulates imiquimod-induced MMP-9 in alveolar macrophages.

### NF-κB Signaling Regulates IAV- and TLR7-Mediated MMP-9 Production in Macrophages

**Figure 3:**
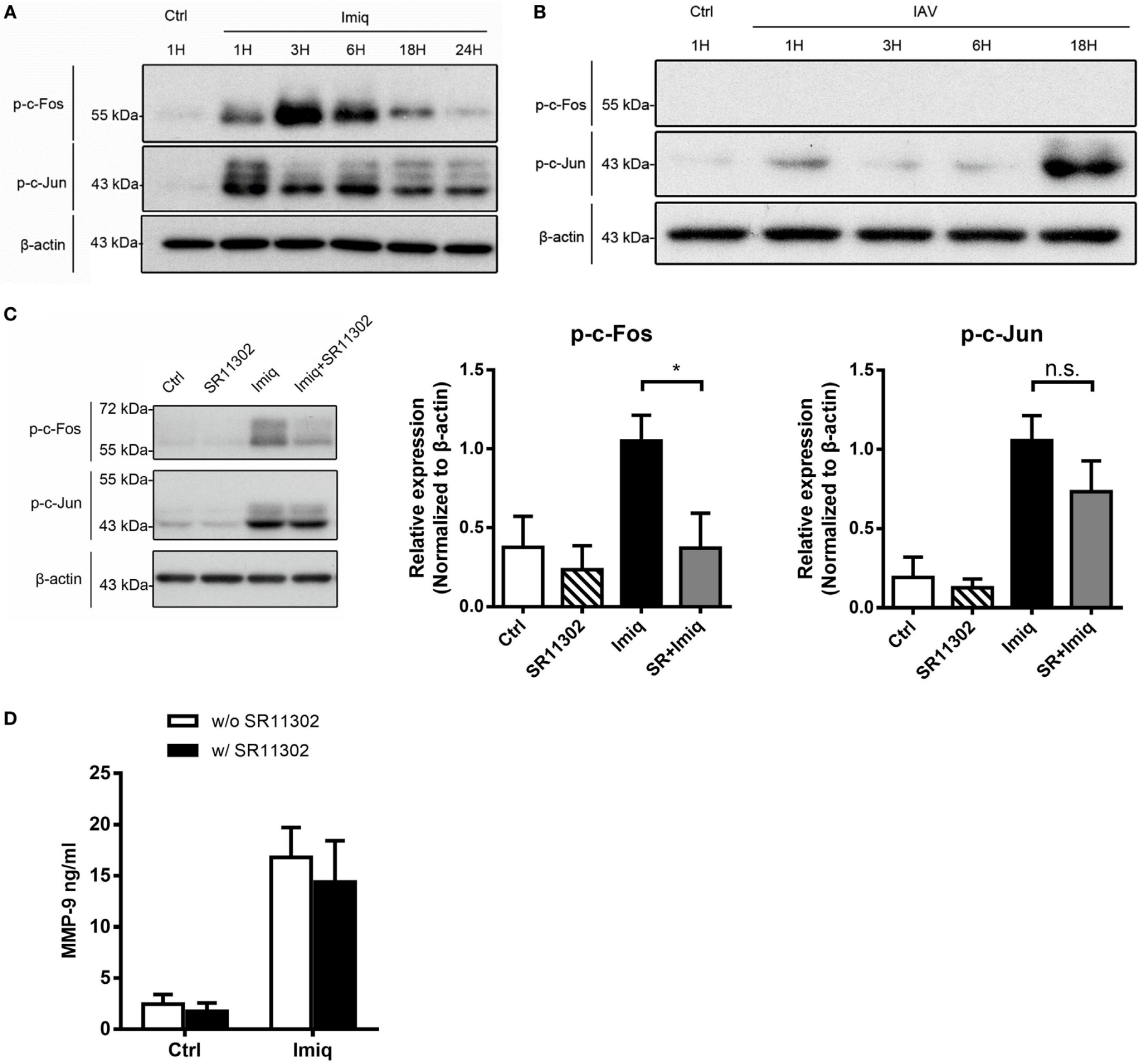
Alveolar macrophage matrix metalloproteinase-9 (MMP-9) production is independent of activator protein 1 (AP-1). MH-S cells were stimulated **(A)** with or without imiquimod (Imiq, 1 µg/ml) and **(B)** WSN virus (MOI = 0.25) for indicated periods of time. **(C)** MH-S cells were pretreated with or without SR11302 (AP-1 inhibitor, 10 µM) for 1 h before stimulation with or without Imiq (1 µg/ml) for another 3 h. **(A–C)** Phosphorylated c-Fos and c-Jun in cell lysate were detected by Western blotting [Panel **(A)**, *n* = 3; Panel **(B)**, *n* = 3; Panel **(C)**, *n* = 6. One representative experiment is shown]. Bar graphs show the levels of indicated protein normalized against β-actin. **(D)** MH-S cells were pretreated with or without SR11302 (AP-1 inhibitor, 10 µM) for 1 h before stimulation with or without Imiq (1 µg/ml) for another 18 h. The concentration of MMP-9 in culture supernatants was quantified by ELISA (*n* = 3, three independent experiments). n.s., not significant, *p < 0.05, compared to control. **(C,D)** were analyzed by one-way ANOVA followed by **(C)** Kruskal–Wallis and **(D)** Sidak’s *post hoc* tests.

**Figure 4:**
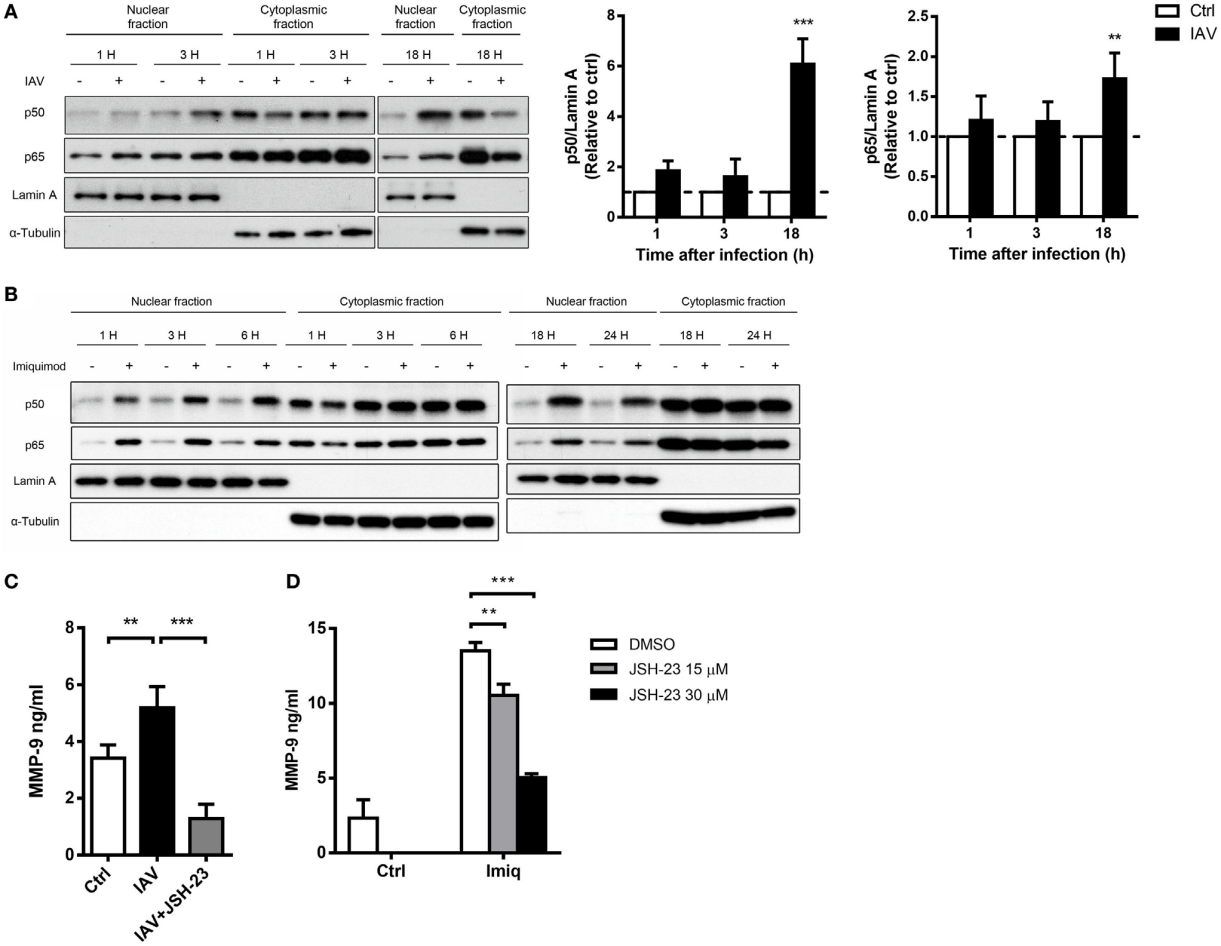
Influenza A virus and toll-like receptor 7 agonist-induced matrix metalloproteinase-9 (MMP-9) production is NF-κB-dependent. MH-S cells were stimulated with or without **(A)** WSN virus (MOI = 0.25) and **(B)** imiquimod (Imiq, 1 µg/ml) for indicated periods of time. Cell lysates were separated into nuclear and cytoplasmic fractions and blotted with anti-p50 and anti-p65, anti-Lamin A (nuclear loading control) and anti-α-tubulin (cytoplasmic loading control) antibodies. One representative experiment is shown in **(A,B)**. Bar graphs in **(A)** show the levels of p50 and p65 protein normalized against Lamin A (*n* = 3, three independent experiments). MH-S cells were stimulated with or without **(C)** WSN virus (MOI = 0.25) and **(D)** Imiq (1 µg/ml) in the presence of DMSO vehicle control or JSH-23 (NF-κB inhibitor, 30 µM) for **(C)** 48 h and **(D)** 24 h [Panel **(C)**, *n* = 5; Panel **(D)**, *n* = 3]. MMP-9 concentration was quantified by ELISA. Data presented are the mean + SD; n.s., not significant, **p < 0.01 and ***p < 0.001 compared to control [one-way ANOVA followed by **(A)** Sidak’s and **(C,D)** Tukey’s *post hoc* test].

To delineate whether TLR7-mediated MMP-9 production is downstream of NF-κB and/or AP-1 signaling pathway, we analyzed NF-κB and AP-1 activation in cells stimulated with imiquimod. Results showed that both c-Fos and c-Jun phosphorylation occurred as early as 1 h after stimulation with imiquimod (Figure 3A). IAV infection induced c-Jun but not c-Fos phosphorylation and the peak of phosphorylation occurred at 18 h after infection (Figure 3B). While chemical inhibition (by SR11302) of AP-1 efficiently reduced the level of phosphorylated c-Fos (Figure 3C), it did not affect MMP-9 production (Figure 3D). Stimulation by either WSN virus or imiquimod significantly induced p50 and p65 nuclear translocation although they followed different kinetics (Figures 4A,B). Inhibition of NF-κB activation by JSH-23 significantly reduced IAV- and imiquimod-induced MMP-9 production (Figures 4C,D). These results show that IAV- and imiquimod-induced macrophage MMP-9 production is NF-κB-dependent.

### CYLD but Not RelB Nor Regnase-1 Is Reduced by MALT1 Activity in Macrophages

**Figure 5:**
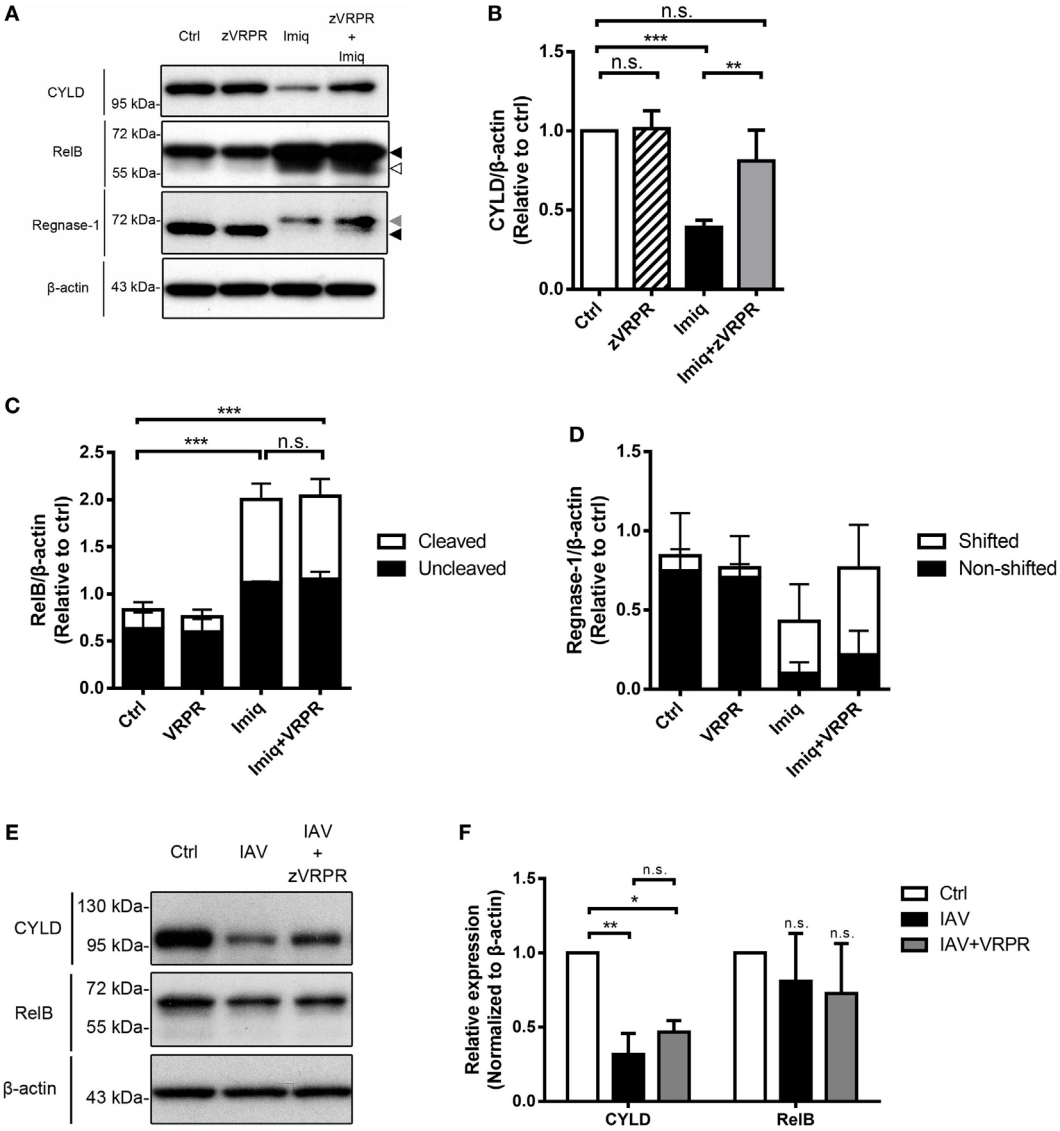
Toll-like receptor 7 signaling and IAV reduce the level of CYLD in alveolar macrophages. **(A)** MH-S cells were pretreated with or without z-VRPR-fmk (zVRPR, 100 µM) for 6 h before stimulation with or without imiquimod (Imiq, 1 µg/ml) for another 18 h. Cell lysates were analyzed by Western blotting with antibodies against CYLD, RelB, and regnase-1. Bar graphs show the levels of **(B)** CYLD, **(C)** RelB, and **(D)** Regnase-1 protein normalized against β-actin (*n* = 4, four independent experiments). Dark arrowhead points to total form of RelB or regnase-1, gray arrowhead to shifted band of regnase-1, empty arrowhead to cleavage form of RelB. **(E)** MH-S cells were pretreated with or without z-VRPR-fmk (zVRPR, 100 µM) for 6 h before stimulation with or without WSN virus (MOI = 0.25) for another 18 h. Cell lysates were analyzed by Western blotting with antibodies against CYLD and RelB. Bar graphs in **(F)** show the levels of CYLD and RelB protein normalized against β-actin (*n* = 3, three independent experiments). One representative experiment is shown in **(A,E)**. **(B–D,F)** Relative intensity was calculated by dividing the intensity of indicated protein by β-actin and normalized against that of control (Ctrl). Data presented are the mean + SD; n.s., not significant; *p < 0.05, **p < 0.01, and ***p < 0.001 compared to control (one-way ANOVA followed by Tukey’s *post hoc*).

In lymphoid cells, MALT1 functions to cleave substrates, thereby regulates NF-κB and/or AP-1 activation (29). MALT1 cleaves regnase-1, which is an RNase that destabilizes mRNAs in response to immune activation in CD4 T cells (30). To investigate the effect(s) of MALT1 on NF-κB activation in alveolar macrophages, we studied CYLD (negative regulator of NF-κB), RelB (transcription factor of non-canonical NF-κB), and regnase-1 degradation in MH-S cells after imiquimod treatment in the presence or absence of MALT1 inhibitor. The level of CYLD was significantly reduced after imiquimod stimulation (Figures 5A,B). Stimulation with imiquimod increased the total and cleavage forms of RelB (Figures 5A,C) and induced rengase-1 mobility shift (Figures 5A,D), which indicates that regnase-1 has been phosphorylated and then degraded (25). Interestingly, however, z-VRPR-fmk treatment reversed only the level of CYLD reduction but did not affect RelB cleavage or mobility shift of regnase-1 protein (Figures 5A–D). Infection by WSN virus, similar to treatment with imiquimod, reduced the level of CYLD but not that of RelB (Figures 5E,F). Inhibition of MALT1 activity by z-VRPR-fmk reversed CYLD degradation slightly but the difference did not reach statistical significance (Figures 5E,F). Our results indicate that MALT1 activation by either imiquimod or IAV reduces the levels of CYLD and that imiquimod-induced reduction is MALT1 activity-dependent.

### Enhanced MMP-9 Production in IAV-Infected Mice Is MALT1-Dependent

**Figure 6:**
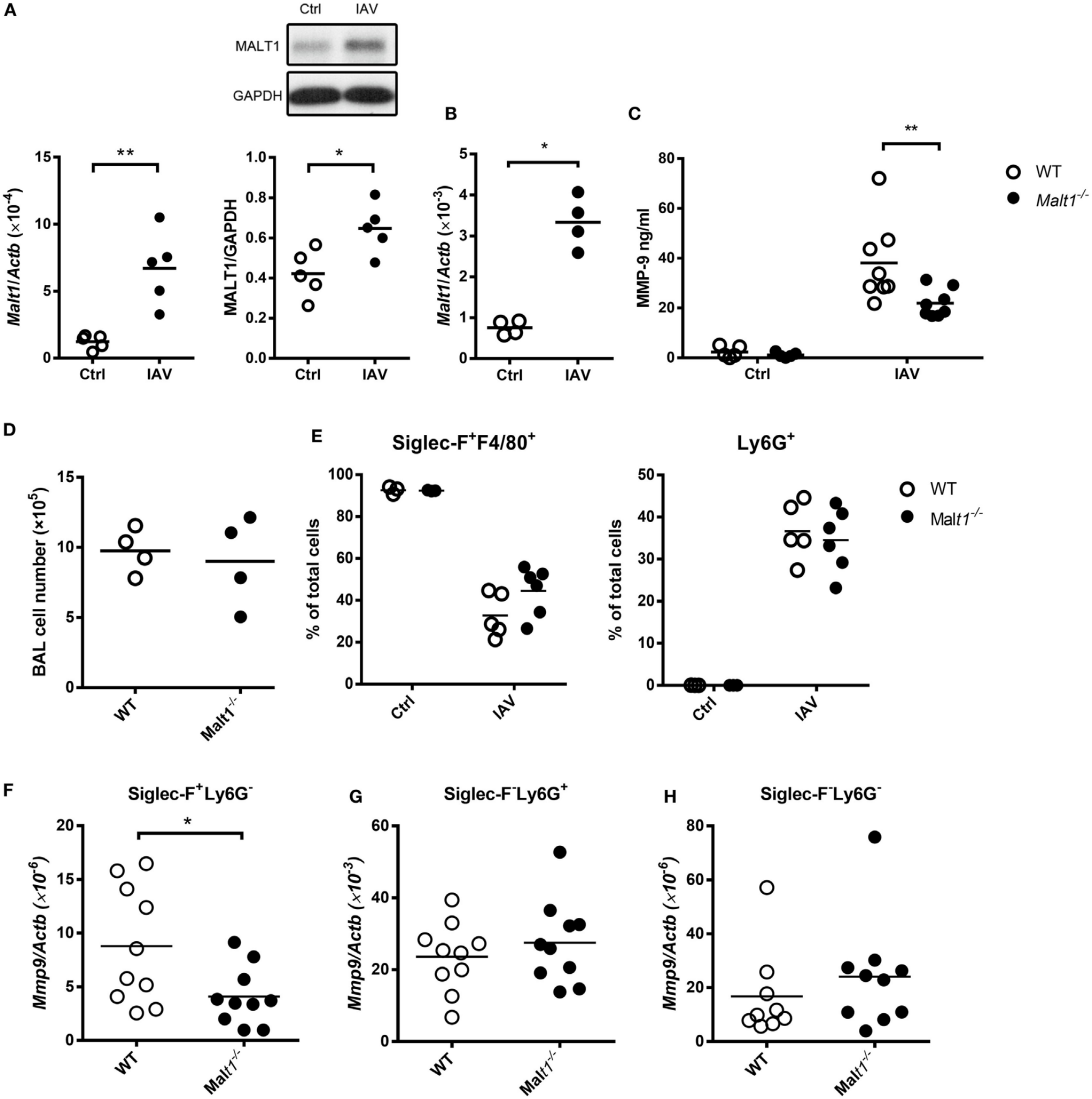
Influenza A virus infection induces mucosa-associated lymphoid tissue lymphoma translocation protein 1 (MALT1) upregulation that contributes to matrix metalloproteinase-9 (MMP-9) production in the lungs. **(A)** Wild-type mice were infected with of 5 × 10^4^ PFU of WSN virus intratracheally. Lungs were harvested from uninfected and infected mice on day 2 after infection. Total RNA in lungs were extracted and applied to qPCR. The mRNA levels of *Malt1*was normalized against *Actb* (left panel). Lysates were blotted with anti-MALT1 and anti-GAPDH antibodies (upper right panel) and the intensity of MALT1 was normalized against that of GAPDH (lower right panel). One representative experiment is shown. **(B–H)** Malt1*^−/−^* and/or wild-type mice were infected with 5 × 10^3^ PFU of WSN virus intratracheally. **(B,D)** On day 4 after infection, bronchoalveolar lavage (BAL) cells were **(B)** subject to RNA extraction for the determination of the levels of *Malt1* transcripts by qPCR and **(D)** counted for the number of cells. **(C,E–H)** BAL was collected on day 2 after infection. **(C)** The concentration of MMP-9 in BAL was quantified by ELISA. **(E)** Cells in BAL were stained with anti-Siglec-F, anti-F4/80, and anti-Ly6G antibodies. Cells were analyzed by flow cytometry. **(F–H)** Cells in BAL were sorted by their expressions of Siglec-F and Ly6G surface markers. Total RNA of sorted cells were extracted and *Mmp9* mRNA were quantified by qPCR and normalized against *Actb*. Each dot represents cells from one mouse. Data are a compilation of two **(A,B,D)** and three **(C,E–H)** independent experiments. *p < 0.05 and **p < 0.01 compared to control. Data in **(A,B,D,F–H)** were analyzed by Mann–Whitney *U*-test. Data in **(C,E)** were analyzed by one-way ANOVA followed by Sidak’s *post hoc* test.

We studied the effect of MALT1 on MMP-9 production by infecting mice with IAV intratracheally. Figure 6A shows that IAV infection upregulated the levels of *Malt1* transcripts and MALT1 protein in the lungs. *Malt1* transcripts were clearly upregulated in cells collected from BAL (Figure 6B). While MALT1 deficiency significantly reduced the level of MMP-9 in BAL fluid after IAV infection (Figure 6C), it did not affect the total cell number nor the percentages of Siglec-F^+^F4/80^+^ and Ly6G^+^ cell populations in BAL (Figures 6D,E). It is worth noting that both macrophages and neutrophils composed of the major cell populations in BAL, and they shared almost equal contribution (35–40%) and MALT1 deficiency did not affect their composition (Figures 6D,E). MALT-1 deficiency reduced the levels of *Mmp9* transcripts in alveolar macrophages (Siglec-F^+^Ly6G^−^) (Figure 6F) but did not affect the levels of *Mmp9* in neutrophils (Siglec-F^−^Ly6G^+^) (Figure 6G) and other cells (Siglec-F^−^Ly6G^−^) (Figure 6H). These data together show that the difference in MMP-9 levels observed in Figure 6C was most likely not due to cellular infiltration but to the differential abilities of MALT1-deficient and WT alveolar macrophages in MMP-9 production (Figures 6D,F). These results highlight the importance of MALT1 in regulating macrophage MMP-9 production during IAV infection.

### MALT1-Deficient Mice Sustain Less Disease Severity after IAV Infection

**Figure 7:**
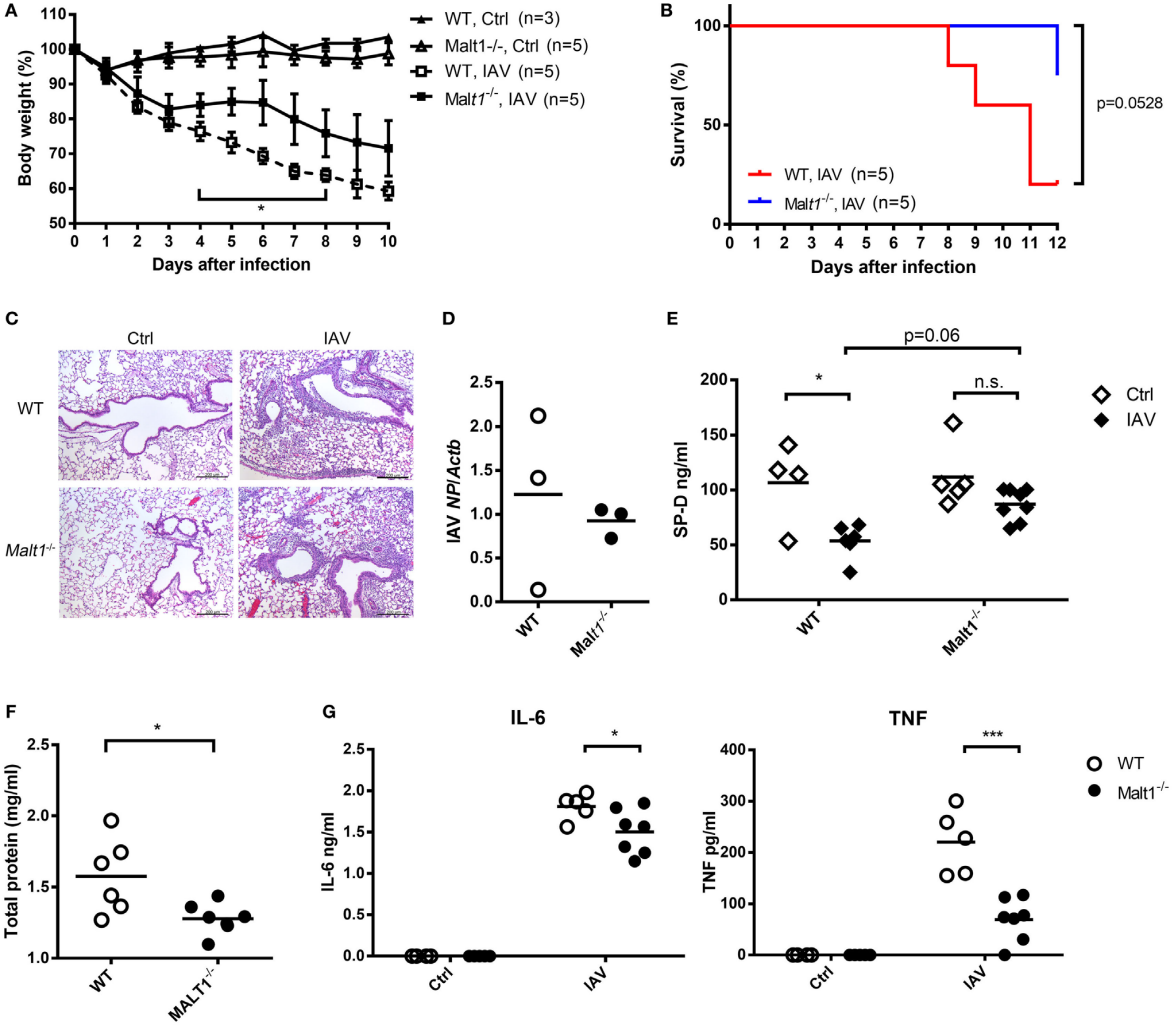
*Malt1^−/−^* mice sustain less disease severity after IAV infection. **(A,B)** Wild-type (WT) and *Malt1^−/−^* mice were infected with or without 1.5 × 10^4^ PFU of HKx31 of IAV intratracheally [*n* as indicated in panels **(A,B)**]. Body weight and survival were recorded daily. **(C–G)** Wild-type and *Malt1^−/−^* mice were infected with or without 5 × 10^3^ PFU of WSN virus. **(C)** Lungs were collected on day 4 after infection and fixed by formaldehyde and stained with hematoxylin and eosin stain (uninfected WT and *Malt1^−/−^* Ctrl, *n* = 2 each; WSN-infected WT and *Malt1^−/−^* mice, *n* = 3 each. One representative experiment is shown). **(D)** Total RNA of lungs was extracted on day 4 after infection and nucleoprotein of IAV mRNA was quantified by qPCR and normalized against *Actb*. **(E–G)** Bronchoalveolar lavage was collected from mice on day 4 after infection. The concentrations of **(E)** surfactant protein D (SP-D) and **(G)** IL-6 and TNF were quantified by ELISA. **(F)** Total protein was determined by Bradford protein assay. **(D–G)** Each dot represents sample from one mouse. n.s., not significant, *p < 0.05, and ***p < 0.001 compared to control. **(A)** Comparisons between IAV-infected WT and *Malt1^−/−^* at the same time point and data in **(D,F)** were analyzed by Mann–Whitney *U*-test. Data in **(B)** were analyzed by log-rank (Mantel–Cox) test. Data in **(E,G)** were by one-way ANOVA followed by **(E)** Tukey’s and **(G)** Sidak’s *post hoc* test.

Wild-type and MALT1-deficient mice were infected with IAV and their body weights and survival were monitored. MALT1-deficient mice had significantly less body weight loss (Figure 7A) and better survival (Figure 7B) than WT mice after IAV infection. While there was no obvious difference in histopathology (Figure 7C) and viral loads (Figure 7D) between MALT1-deficient and WT mice. MALT1-deficient mice had significantly higher levels of SP-D (Figure 7E) and lower levels of total protein (Figure 7F) in BAL than WT mice. Additionally, the levels of IL-6 and TNF were significantly lower in MALT1-deficient than in WT mice (Figure 7G). As low SP-D levels and high total protein in BAL fluid correlate with lung injury (31–33), our results show that MALT1 is involved in controlling lung injury during IAV infection.

## Discussion

Innate immune cells act as sentinels standing on the first line to respond quickly to infections (27). However, the robust innate immune activities triggered by pathogen-associated molecular patterns that detect invading IAV not only facilitate viral elimination but in the meantime also result in immune-mediated tissue injury (27). We and others have shown that MMP-9 is associated with tissue pathology in a pulmonary IAV infection mouse model (8, 9). It is shown that IAV infects not only lung epithelial cells but also macrophages (34). In this study, we showed that alveolar macrophages produce MMP-9 as a result of triggering TLR7 signaling pathway and IAV stimulation. MMP-9 response in alveolar macrophages is positively modulated by MALT1. MALT1 deficiency results in reduced MMP-9, TNF, and IL-6 levels and less severe disease after IAV infection. As alveolar macrophages are important to host response to infection and MMP-9 plays a role in tissue injury (9), regulation of the production of MMP-9 by macrophages has significant implication in pulmonary infections.

Bradley et al. reported that neutrophils in lungs of IAV-infected mice produce MMP-9 (10). Here, we discovered that alveolar macrophages, in addition to neutrophils, are MMP-9 producers. Macrophage and neutrophil each constitute about 35–40% of the total cell population in BAL. Although the level of *Mmp9* transcripts in neutrophils is higher than in macrophages, without the knowledge of the efficiencies of protein translation in neutrophils and macrophages, it remains to be determined the relative contribution of neutrophil and macrophage populations in production of MMP-9 protein in the lungs. Macrophages are one of the major cell populations and MMP-9 producers in the lungs. While neutrophil production of MMP-9 is not regulated, our results support the notion that regulation of macrophage MMP-9 production by MALT1 has impact on IAV-induced lung injury. It is reported that MMP-9 is involved in tissue remodeling and regulation of the activity of inflammatory mediators (3, 35). Thus, it is possible that through production of MMP-9, macrophages play a pathogenic role in IAV infection.

Both NF-κB and AP-1 have been reported to be involved in MMP-9 production. Stimulation of THP-1 cells by heat-killed *Listeria monocytogenes* induces both AP-1 and NF-κB activation and inhibition of NF-κB activity reduces MMP-9 production (19). Human umbilical vein endothelial cells stimulated with fibronectin and vitronectin produce MMP-9 and silencing AP-1 suppresses MMP-9 production (36). We used chemical inhibitors at a concentration not toxic to cells to show that MMP-9 production by macrophages stimulated with TLR7 agonist and IAV is NF-κB dependent (Figure S1 in Supplementary Material; Figures 4C,D). Thus, it appears that the involvement of NF-κB and AP-1 in MMP-9 production is dependent on the type of cells and stimuli.

Mucosa-associated lymphoid tissue lymphoma translocation protein 1 regulates NF-κB signaling through its scaffold and paracaspase functions. Scaffold function of MALT1 mediates CBM complex formation and through its paracaspase function, MALT1 degrades NF-κB negative regulators (22). Paracaspase activity of MALT1 is important for optimal T-cell receptor (TCR)- and B-cell receptor (BCR)-induced NF-κB activation in lymphocytes and for C-type lectin receptor-induced C-Rel activation in dendritic cells (37–39). A20, BCL10, CYLD, RelB, regnase-1, roquin 1/2, and MALT1 itself have been identified to be MALT1 proteolytic substrates upon TCR and BCR activation (30, 37, 39–41). In this study, we demonstrated in macrophages that CYLD is reduced after stimulation by TLR7 agonist and IAV. The reduction of CYLD after stimulation by TLR7 agonist is MALT1 activity-dependent. Interestingly, stimulation with TLR7 agonist also induces RelB cleavage and change of the mobility of regnase-1 protein, but these changes are MALT1 activity independent. Work was undertaken to investigate whether A20 is also an MALT1 substrate in macrophages upon stimulation by TLR7 agonist. However, commercially available anti-A20 antibody did not recognize murine A20 (data not shown) or its cleavage form (42). The result was inconclusive. It appears that in macrophage response to TLR7 stimulation for MMP-9 production, paracaspase MALT1 preferentially uses CYLD as a substrate. Whether activation of MALT1 in other myeloid cells by triggering pattern recognition receptors preferentially use CYLD or other substrates is an interesting question needs to be addressed.

A recent report by Gewies et al. showed the comparison of *Malt1^−/−^* mice to paracaspase mutant MALT1 mice (*Malt1*^PM^) (42). Animal growth and viability in homeostatic conditions are not affected by the absence of MALT1, yet, *Malt1*^PM^ mice develop severe cachexia with increased numbers of T and B cells in the lymph nodes (42). These results suggest that scaffold and paracaspase functions of MALT1 are responsible for distinct cellular functions. In the present study, we show that MALT1 deficiency or inhibiting MALT1 activity suppresses TLR7-mediated MMP-9 production in macrophages. These results clearly indicate that MALT1 activity is important to the regulation of MMP-9 production but whether MALT1 functions as a scaffold in macrophages still remains to be clarified.

In addition to MMP-9, we also observed that TNF and IL-6 are reduced in BAL of MALT1-deficienct mice after IAV infection. TNF concentration in BAL is associated with fever and pulmonary lesion during IAV infection (43). Anti-TNF antibody treatment reduces body weight loss and illness score compared to untreated mice after IAV infection (44). Since TNF is downstream of NF-κB signaling (45) and MALT1 is a positive regulator of NF-κB signaling (20), it is possible that MALT1 regulates not only the production of MMP-9 but also that of TNF during IAV infection. Results reported by Haasbach et al. showed that intravenous injection of NF-κB inhibitor ameliorates IAV-induced body-weight loss and viral titer support our speculation (46). It appears that the interplay between NF-κB and the severity of IAV infection is a complex issue. To study the role of MALT1 in regulation of NF-κB-related factors may provide insight to the overall picture of the pathogenesis of IAV infection.

In summary, our study demonstrates for the first time that (i) MALT1 modulates TLR7 agonist- and IAV-induced MMP-9 response in alveolar macrophages; (ii) MALT1 mediates CYLD reduction in macrophages upon TLR7 stimulation; (iii) MMP-9 production in alveolar macrophages is through NF-κB but not AP-1; and that (iv) MALT1 deficiency results in reduced IAV-induced disease severity. Together, our findings point to the novel role of MALT1 in regulating MMP-9 production in alveolar macrophages. These findings offer the possibility of modulating MALT1 to regulate the functions of alveolar macrophage in respiratory viral infection.

## Ethics Statement

This study was approved by the Institutional Animal Care and Use Committee of the National Taiwan University College of Medicine and College of Public Health (IACUC No. 20140314). All experiments were carried out in strict accordance to the Guidebook for the Care and Use of Laboratory Animals, The Third Edition, 2007, published by The Chinese-Taipei Society of Laboratory Animal Sciences (Taipei, Taiwan). All infection experiments in mice were performed following the guidelines of biosafety level 2.

## Author Contributions

Y-HL and BAW-H formed the original concepts and designed experiments. Y-HL, J-HH, and T-HC performed experiments. Y-HL acquired and interpreted data. H-CY provided HKx31 strain of IAV and contributed to experimental design. Y-HL, J-HH, and BAW-H drafted and finalized the submitted manuscript.

## Conflict of Interest Statement

The authors declare that the research was conducted in the absence of any commercial or financial relationships that could be construed as a potential conflict of interest.
