# Supplementary material for: Mucosa-Associated Lymphoid Tissue Lymphoma Translocation Protein 1 Positively Modulates Matrix Metalloproteinase-9 Production in Alveolar Macrophages upon Toll-Like Receptor 7 Signaling and Influenza Virus Infection
Source: Front Immunol. 2017 Sep 22;8:1177. doi: 10.3389/fimmu.2017.01177 (PMC5614920; doi:10.3389/fimmu.2017.01177)
Supplement: Supplementary file 1 [file Data_Sheet_1.pdf]

Supplementary Material

Mucosa-Associated Lymphoid Tissue Lymphoma Translocation Protein 1 Positively Modulates Matrix Metalloproteinase-9 Production in Alveolar Macrophages upon Toll-Like Receptor 7 Signaling and Influenza Virus Infection

Yu-Hsiang Lee, Juin-Hua Huang, Tzu-Hsuan Chang, Hung-Chih Yang, and Betty A. Wu-Hsieh*

*** Correspondence:**

Betty A. Wu-Hsieh

bwh@ntu.edu.tw

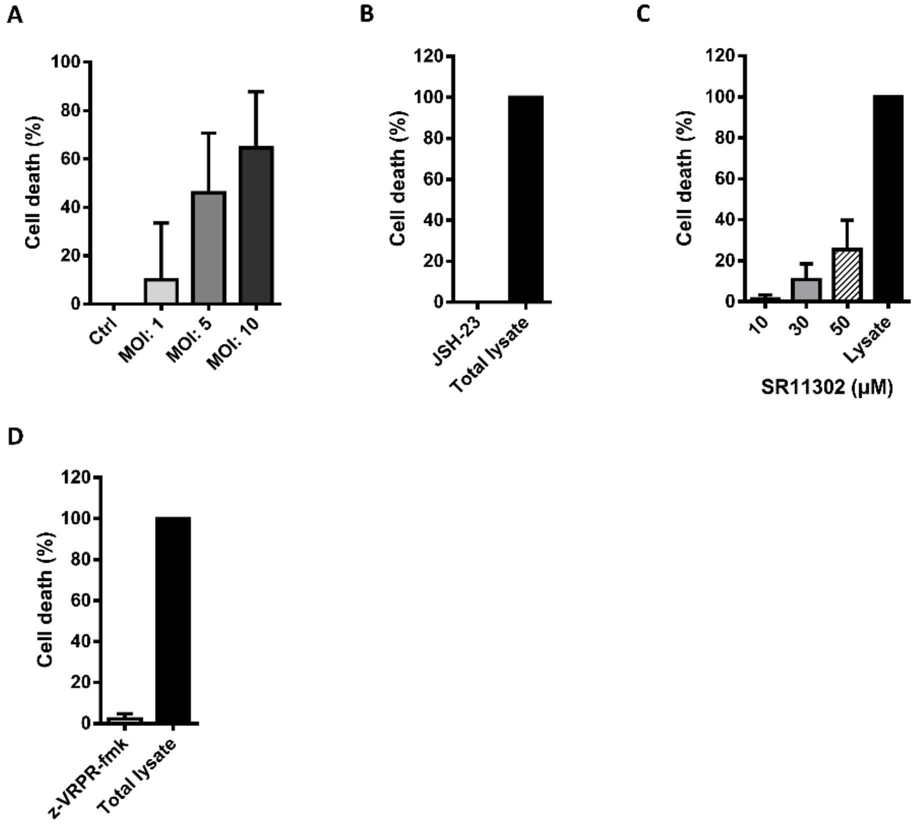

Supplementary Figure 1. Percent cell death of cells cultured in inhibitors and after IAV infection. MH-S cells were treated with **(A)** different MOI of HKx31 virus as indicated for 48 h, **(B)** 30 µM of JSH-23 for 24 h, **(C)** different concentrations of SR11302 for 19 h and **(D)** 100 µM of z-VRPR-fmk for 24 h. Culture medium and total cell lysate were collected after incubation and applied to LDH cytotoxicity assay.
